# The essential roles of protein–protein interaction in sigma-1 receptor functions

**DOI:** 10.3389/fncel.2013.00050

**Published:** 2013-04-23

**Authors:** Mohan Pabba

**Affiliations:** Neurosciences Unit, Department of Cellular and Molecular Medicine, Faculty of Medicine, University of OttawaOttawa, ON, Canada

σ-1R is the well-known subtype of σ-Rs that were originally proposed in 1976 (Martin et al., 1976). σ-1R is a 223 amino acid integral membrane protein consisting of a short N-terminus, a large C-terminus tail, and two transmembrane domains: one at the N-terminus and the other in the middle of the protein (Su et al., 2010). σ-1Rs are distributed throughout the brain. At the subcellular level, σ-1Rs are mainly localized at the endoplasmic reticulum (ER)/mitochondrial associated membranes (MAM) and at very low levels in post-synaptic thickenings of the neuron (Alonso et al., 2000; Su et al., 2010). σ-1Rs at ER/MAM membranes exist in clustered globular structures that are enriched with cholesterol and neutral lipids (Hayashi and Su, 2003, 2005). Several categories of drugs bind to σ-1Rs, for example: cocaine, dihydroepiandrosterone, dimethyl tryptamine (DMT), psychotomimetic compounds, and haloperidol (antagonist). The steroids and DMT were proposed to act as endogenous ligands for the σ-1R. Studies from various laboratories performed on heterologous, *in vivo* and *ex vivo* systems by employing multidisciplinary techniques demonstrated that the σ-1R interacts with numerous cellular components (Su et al., 2010), e.g., different classes of ion channels, kinases, G-protein coupled receptors (GPCRs), etc. The σ-1R associates with voltage-gated ion channels, e.g., Na^+^, K^+^, and Ca^2+^. Interaction of the σ-1R with voltage-gated K^+^ and Ca^2+^ channels results in either inhibition or enhancement in the activities of these ion channels, whereas σ-1R interaction with voltage-gated Na^+^ channels results in inhibition of the channel activity (Kourrich et al., 2012). On the other hand, σ-1R enhances the activity of *N*-methyl-D-aspartate receptors (NMDARs) (a ligand-gated ion channel) and dopamine D_1_ receptors (a GPCR) (Monnet et al., 1990; Navarro et al., 2010). The σ-1R modulation of D_1_R is through protein–protein interactions (Navarro et al., 2010). However, it is yet to be determined whether σ-1R modulates the NMDAR function through protein–protein interactions. Nevertheless, a recent study demonstrated that σ-1R inhibits the activity of small conductance Ca^2+^-activated K^+^-channels (SK channels), and consequently potentiates the NMDAR function (Martina et al., 2007). It is still unknown if there is any physical association between SK channels and σ-1Rs.

**Figure 1:**
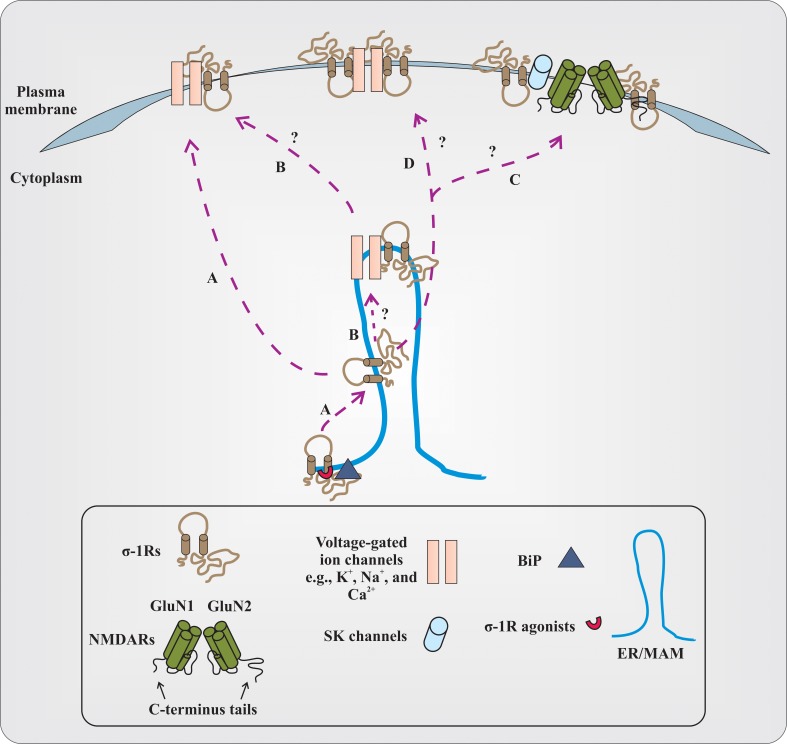
**Proposed and elusive mechanism(s) of σ-1R modulation of the function of voltage-gated and ligand-gated ion channels. (A)** The prevailing hypothesis is that activation of σ-1Rs with high concentrations of their agonists (red colored semi-circle) causes σ-1Rs two transmembrane domains (black colored line boundaries and respective tails in a light brown color), at the ER/MAM, to dissociate from the chaperone called BiP (dark gray colored triangle). The dissociated σ-1Rs translocate to the plasma membrane or plasmalemma and in a subtype dependent manner, either inhibit or enhance the function of ion channels by forming protein-protein interactions. **(B)** It remains to be identified if the dissociated σ-1Rs associate with ion channels at the ER/MAM, and after association, whether or not the entire complex is translocated toward the plasma membrane. The study from Kourrich et al. (2013) supports this possibility, at least with voltage-gated K^+^-channel Kv1.2. **(C)** It is unclear if dissociated and translocated σ-1Rs associate with SK channels or NMDARs. The functional NMDARs are tetrameric assembles of two obligatory GluN1 subunits with either two GluN2 subunits (different combinations of GluN2 subunits) or GluN3 subunits. For representation, the dimer form of NMDARs (GluN1 and GluN2 subunits) and their C-terminus tails are presented in the figure. The N-terminus domain of NMDAR subunits and GluN3 subunits are not shown. **(D)** The other possibilities that remain unexplored are whether the dimers of the σ-1R interact with ion channels, and the stoichiometry of interaction between the σ-1R and ion channels.

How does the σ-1R, being an intracellular protein, modulate the functions of numerous cellular components that are present at the plasma membrane? The prevailing hypothesis is that under resting conditions, at ER/MAM, σ-1Rs are associated with chaperone called BiP. Upon activation of σ-1Rs by their agonists (at concentrations ~ equal to or less than 10 times their *K*_*i*_ value), σ-1R dissociates from BiP and modulates the function of inositol triphosphate (IP3) receptors. The σ-1R modulation of IP3 receptor function consequently affects Ca^2+^ influx and signaling into the mitochondria (Hayashi and Su, 2007). However, if σ-1R agonists are present at high concentrations (~>10 times their *K*_*i*_ value) or during the ER stress, σ-1R dissociates from BiP and translocates to the plasma membrane or plasmalemma and modulates the activities of various cellular components via protein–protein interactions (Su et al., 2010). While this model is promising, several outstanding questions remain to be addressed with respect to σ-1Rs and their association with cellular components, especially different classes of ion channels (Figure 1). For instance, first, it needs to be clarified whether the σ-1R modulates multiple cellular components at the plasma membrane or at the plasmalemma of neurons (Su et al., 2010). Second, it is unclear at the moment if the σ-1R associates with ion channels (e.g., voltage-gated Na^+^ or K^+^ channels) at the ER/MAM, and after association, whether or not the entire complex (σ-1R-ion channel) is translocated to the plasma membrane. Third, investigations from different laboratories demonstrated that the treatment of animals with several σ-1R ligands alter the behavior of animals in various behavioral paradigms such as cocaine-induced behavioral response, NMDAR-antagonism induced amnesia, etc. Hence, it remains to be investigated if there is any link between σ-1Rs association with voltage-gated and/or ligand-gated ion channels, and the alteration in animal behavior, at least in the above-mentioned conditions. Forth, how the σ-1R modulates the functions of voltage-gated and ligand-gated ion channels heterogeneously and to a varying degree remains elusive. Although there can be multiple factors involved, the following reasons could play an important role in differential regulation of voltage-gated and ligand-gated ion channels by σ-1Rs. (a) Recently, it is shown that the σ-1R could exist in dimers (Chu et al., 2013); therefore, do the dimers of σ-1R associate with ion channels? (b) What is the stoichiometry of the σ-1R interaction with ion channels? (c) Several lines of evidence demonstrate that there exist subtypes within the σ-1Rs (Bergeron and Debonnel, 1997; Shioda et al., 2012); thus, do these subtypes display differences in association with ion channels? (d) What is the conformational crystal structure of the σ-1R in association with either voltage-gated or ligand-gated ion channels? (e) Which amino acid residues or motif(s) in the σ-1R determine the σ-1R association with various classes of ion channels? (f) Which polar and non-polar amino acid residues of the σ-1R influence the biophysical properties of σ-1R-associated ion channels? The crystallographic data could provide enough evidence regarding the residues and domains of the σ-1R involved in physical association with ion channels. Additionally, it could also provide information regarding the differences in associations between σ-1Rs and various ion channels, if there are any. Finally, are there any spatial requirements for the σ-1R to associate with ion channels, given σ-1R-modulated ion channels (e.g., voltage-gated Na^+^ and K^+^ channels) have some overlapping distribution in the neuron (Trimmer and Rhodes, 2004)?

However, a recent study by employing a multidisciplinary approach elegantly demonstrated that the σ-1R's interaction with voltage-gated K^+^ channel Kv1.2 plays an important role in the cocaine-induced locomotor sensitization (Kourrich et al., 2013). Using pharmacological blockers and knockdown of σ-1Rs, Kourrich et al. (2013) first identified that σ-1Rs are involved in cocaine-induced long-lasting neuronal and behavioral adaptation. Then, by employing electrophysiological recordings as well as biochemical studies on *ex vivo* tissue and heterologous cells, they confirmed that the σ-1R's interaction with voltage-gated K^+^ channel Kv1.2 contributes/participates in the behavioral response (locomotor sensitization) to cocaine. Studies from Kourrich et al. (2013) have provided valuable information in terms of the σ-1R's association with ion channels, at least with voltage-gated K^+^-channels such as: (a) the σ-1R could possibly associate with these ion channels at the ER/MAM, and then the entire complex is trafficked toward the plasma membrane; (b) the potential link between σ-1R association with these ion channels and alteration in the behavior of animals; and importantly, (c) the structural orientation of the σ-1R at the plasma membrane during the association with these ion channels, for example, both N-terminus and C-terminus tails of the σ-1R are extracellular. Additionally, this study also provided first direct and unequivocal evidence for the presence of σ-1Rs at the plasma membrane. Nonetheless, even though Kourrich et al. (2013) demonstrated that both N-terminus and C-terminus tails of the σ-1R are extracellular, the orientation of the σ-1R *in vivo* needs to be confirmed. Also, future studies are necessary to test if the σ-1R's interaction with other ion channels alters the behavior of animals similar to what is shown by Kourrich et al. (2013). For example, is there any interaction between σ-1Rs and NMDARs that could ameliorate the behavioral phenotype observed in mouse models of amnesia induced by the blockade of NMDARs (Maurice et al., 1994)?

It is essential to identify and understand the molecular details of the σ-1R association with cellular components (Su et al., 2010) such as different classes of ion channels, kinases, GPCRs, etc. Gaining substantial knowledge on the details about σ-1Rs and their structural determinants responsible for association with ion channels as well as other cellular components could help decipher the σ-1R's role in pathological conditions such as addiction, amnesia, frontotemporal degeneration in motor neuron disease (FTLD-MND), etc. (Hayashi et al., 2011). Furthermore, insights on molecular features of the σ-1R interactions with various classes of ion channels provide the opportunity to understand the critical role of σ-1Rs in neuronal plasticity (Su et al., 2010; Kourrich et al., 2012). Defining the intricacies underlying the relationship between σ-1Rs and their interacting partners during physiological and pathological conditions will form a venue for the development of novel therapeutic strategies.
